# Mask Gradient Response-Based Threshold Segmentation for Surface Defect Detection of Milled Aluminum Ingot

**DOI:** 10.3390/s20164519

**Published:** 2020-08-12

**Authors:** Ying Liang, Ke Xu, Peng Zhou

**Affiliations:** 1 Collaborative Innovation Center of Steel Technology, University of Science and Technology Beijing, Beijing 100083, China; liangyinghero@gmail.com; 2 Research Institute of Artificial Intelligence, University of Science and Technology Beijing, Beijing 100083, China; zhoupeng@nercar.ustb.edu.cn

**Keywords:** surface inspection, aluminum ingot, mask gradient response, Difference of Gaussian, inception-v3

## Abstract

The surface quality of aluminum ingot is crucial for subsequent products, so it is necessary to adaptively detect different types of defects in milled aluminum ingots surfaces. In order to quickly apply the calculations to a real production line, a novel two-stage detection approach is proposed. Firstly, we proposed a novel mask gradient response-based threshold segmentation (MGRTS) in which the mask gradient response is the gradient map after the strong gradient has been eliminated by the binary mask, so that the various defects can be effectively extracted from the mask gradient response map by iterative threshold segmentation. In the region of interest (ROI) extraction, we combine the MGRTS and the Difference of Gaussian (DoG) to effectively improve the detection rate. In the aspect of the defect classification, we train the inception-v3 network with a data augmentation technology and the focal loss in order to overcome the class imbalance problem and improve the classification accuracy. The comparative study shows that the proposed method is efficient and robust for detecting various defects on an aluminum ingot surface with complex milling grain. In addition, it has been applied to the actual production line of an aluminum ingot milling machine, which satisfies the requirement of accuracy and real time very well.

## 1. Introduction

Surface defect detection is a critical step of the metal industry. Since the technologies under development are becoming more and more feasible, and the results are reliable enough for a decision, the optical non-destructive testing (ONDT) has gained more and more attention in this filed. This is mainly due to the development of the used tools: laser, cameras, and those faster computers that are capable of processing large amounts of encrypted data in optical measurements [1]. A review has been provided in [2], which is about the main ONDT technologies, including fiber optics [3], electronic speckle [4], infrared thermography [5], endoscopic, and terahertz technology. The focus of this paper is the digital speckle measurement method because of the use of CCD technology and advanced computer vision technologies. For the high-quality inspection of various types of materials in all kinds of environments, the advanced computer vision technologies have evolved into a mainstream and replaced the conventional manual inspection method, improving on its inefficiency and high labor intensity.

**Figure 1:**
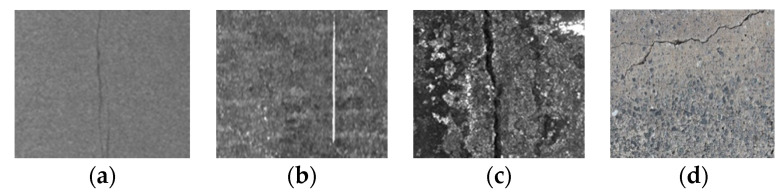
Statistical textures examples. (**a**) Hot-rolled steel strips surface, homogeneous; (**b**) Con-casting slabs surface, homogeneous; (**c**) Con-casting slabs surface, inhomogeneous; (**d**) Bridge deck inhomogeneous.

**Figure 2:**
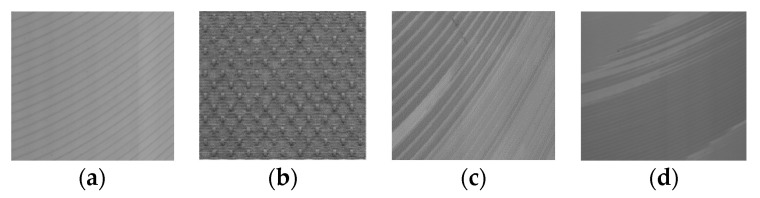
Structural textures examples. (**a**) Milled surface of aluminum ingot, oriented, homogeneous; (**b**) Fabric, isotropic, homogeneous; (**c**,**d**) Milled aluminum ingot surface, inhomogeneous.

Texture analysis provides a very powerful tool to detect defects in applications for visual inspection, since textures provide valuable information about the features of different materials [6]. In computer vision, texture is broadly divided into two main categories: statistical and structural. As shown in Figure 1, statistical textures are isotropic and do not have easily identifiable primitives. In contrast, structural (or patterned) textures are characterized by a set of repetitive primitives and placement rules, as shown in Figure 2. Both the statistical and structural textures appear as homogeneous (Figure 1a,b and Figure 2a,b) or inhomogeneous (Figure 1c,d and Figure 2c,d). It should be noted that Figure 1d and Figure 2b are respectively quoted from Reference [7] and Reference [8]. As can been seen, the milling surface we deal with features structured homogeneous or inhomogeneous textures (Figure 2a,c,d).

In order to enable automatic and non-destructive detection, visual inspection systems have found wide applications in surface detection such as concrete structures [7,9,10,11] and metal surfaces [12,13,14,15,16,17,18,19,20,21,22,23,24,25,26,27,28,29,30]. In the field of concrete structure, there are lots of studies that try to inspect cracks from image analysis [7,9,10,11]. In the field of metal surfaces, visual inspection systems have been applied in both ferrous metal and nonferrous metal surface detection. For the nonferrous metals, methods to detect the surface defects of various products such as aluminum strips [12,13,14], aluminum foils [15], and aluminum profiles [16,17,18] have been well established. About the ferrous metal, the types of steel surfaces studied for defect detection based on vision include slab [14,19,20], plate [21,22,23], hot strip [24,25,26], and cold strip [27,28,29]. The comprehensive survey for typical flat steel products can be found in [30]. In general, the above defect detection techniques can be roughly divided into three categories: statistical, filtering, and machine learning.

The statistical method is to establish a mathematical model using probability theory and mathematical statistics, which can be used to infer, predict, quantitatively analyze, and summarize the spatial distribution data of pixels [31]. Reference [7] presents a multiple features-based cracks detection algorithm of bridge decks. A comprehensive analysis of multiple features (intensity-based, gradient-based, and scale-space) and multiple classifiers (random forests, support vector machines, and adaboost) show a peak classifier performance of 95%. Reference [24] proposed a simple yet robust feature descriptor against noise named the adjacent evaluation completed local binary patterns for hot-rolled steel strip surface defects recognition. Filtering-based methods commonly apply a filter bank to an image to calculate the energy of the filter response. To provide an efficient multi-scale directional representation of different defects, the shearlet transform is introduced in [14]. With the popularity of artificial intelligence in recent years, machine learning has been applied extensively in surface defect detection. Reference [9] used a supervised machine learning method called light gradient boosting machine (LightGBM) to detect cracks from the concrete surface imagery. The features are derived from pixel values and geometric shapes of cracks. In addition, spectral filtering approaches are suitable for the defect detection of uniform textured images composed of basic texture primitives with a high degree of periodicity [32]. Fourier transform (FT) was used in [33] to detect defects in directionally textured surfaces. Nevertheless, the FT-based approaches are inadequate under the circumstances that Fourier frequency components related to the background and defect areas are highly mixed together [34]. Gabor wavelet was used in [30] to extract features of images with periodic texture. Wavelet transform has been successfully applied in defect detection on statistical surfaces such as cold-rolled steel strips [27] and hot-rolled steel strips [35], and it has also been well used for homogeneous patterned surfaces [36]. Navarro et al. [6] present a wavelet reconstruction scheme to detect defects in a wide variety of structural and statistical textures.

Recently, fine-designed deep convolutional neural networks have emerged as powerful tools in a variety of computer vision tasks. Reference [10] proposed an improved You Only Look Once (YOLOv3) with transfer learning, batch renormalization, and focal loss for concrete bridge surface damage detection. The improved single-stage detector achieved a detection accuracy of 80% on a dataset containing a total of 2206 inspection images labeled with four types of concrete damages. Reference [11] proposed a crack detection method based on deep fully convolutional network (FCN) semantic segmentation with the VGG16 backbone on concrete crack images. The FCN network is trained end-to-end on a subset of 500 annotated 227 × 227-pixel crack-labeled images and achieves about 90% in average precision. An end-to-end steel strip defect detection network model was outlined in [28]; this system is based on the symmetric surround saliency map for surface defects detection and deep convolutional neural networks (CNNs) for seven classes of steel strip defects classification. To inspect the defects of a steel surface, Reference [23] presents a new classification priority network (CPN) and a new classification network, multi-group convolutional neural network (MG-CNN).

**Figure 3:**
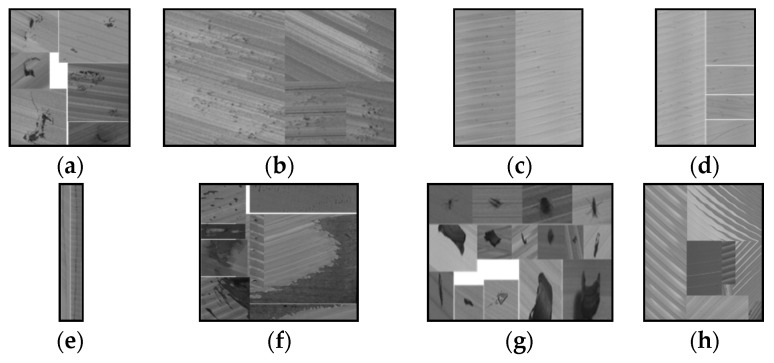
Samples of different defects: (**a**) Slag inclusion (SI); (**b**) Pitted slag inclusion (PSI); (**c**) Adhesion aluminum (AA); (**d**) Scratches (Sc); (**e**) Crack (Cr); (**f**) Oxide film (OF); (**g**) Mosquito (Mo) and aluminum chips (AC); (**h**) Texture background (Tb).

However, these defect detection methods are primarily used for only crack defects on concrete structures or metal surfaces with non-texture backgrounds. As far as we know, there is no literature on the surface defect detection of aluminum ingots with a milling grain background. The surface of aluminum ingot after milling always has multi-directional and multi-scale grinding texture patterns; sometimes, the distribution of the grinding ridge is uneven. After milling, various surface defects (Figure 3) will appear on the surface of aluminum ingot such as small local defects (Figure 3a), distributed defects with complex texture and fuzzy boundaries (Figure 3b-d), longitudinal linear defects throughout the whole picture (Figure 3e), and large-scale distributed defects with irregular shapes (Figure 3f). In addition, there are many pseudo defects with various patterns on the surface of aluminum ingot, such as aluminum chips (AC), mosquito (Mo) (Figure 3g), and the milling grain (Figure 3h). These factors greatly increase the difficulty of defect detection and recognition. To handle these problems, we propose a detection algorithm of aluminum ingot surface defects combining traditional detection and deep learning classification, which has been applied to the production line of an aluminum ingot milling surface.

The main contributions of the paper are summarized below:

1. In terms of ROI extraction in an aluminum ingot image, we design a novel mask gradient response-based threshold segmentation algorithm to iteratively separate out defects of varying significance. In addition, the combination of the mask gradient response-based threshold segmentation (MGRTS) and Difference of Gaussian (DoG) can effectively improve the detection rate of the above defects.
2. In the classification stage, we use the inception-v3 network structure with focal loss in the training process and data augmentation technologies to overcome the class imbalance problem and realize the accurate identification of various defects.
3. Our method can make full use of central processing unit (CPU) and graphics processing unit (GPU) resources in a workstation or server. Even if the server used in the production line is not configured with GPU, the algorithm can still ensure the realization of rapid defect detection.
4. At the beginning of the project, even without a large number of labeled samples, the algorithm can still deploy and detect the suspicious regions quickly owing to the improved ROI detection algorithm.

## 2. Materials and Methods

**Figure 4:**
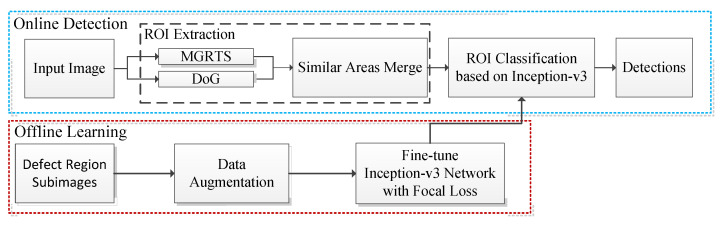
Overview of the defect detection method applied to a real production line. DOG: Difference of Gaussian, MGRTS: mask gradient response-based threshold segmentation, ROI: region of interest.

In this section, the proposed two-stage surface defects detection method will be introduced in detail. Since there is no similar defect database, at the beginning of the project, we need to preliminarily detect the area of interest and collect defect samples. Therefore, we cannot use the end-to-end network which needs a large number of labeled defect images; instead, we design a two-stage target detection method. As shown in Figure 4, the proposed method has two main components: (1) ROI extraction based on the combination of MGRTS, DoG, and similar area merge, and (2) defect ROI classification.

### 2.1. ROI Extraction

In the region of interest (ROI) extraction stage, in order to ensure the detection rate of defects, DOG and edge detection with the MGRTS are used to jointly complete the detection of suspicious areas of defects, and special post-processing is adopted to merge similar areas that may be distributed defects. In the proposed method, edge detection with MGRTS can iteratively segment most of the suspicious regions of defects, while the DoG method is mainly used to detect large-scale defects that cannot be completely segmented by MGRTS, and defects that can be missed by the MGRTS when the background texture gradient is strong.

#### 2.1.1. MGRT-Based Iterative Threshold Segmentation

In the MGRTS, the mask gradient response is the gradient map after the strong gradient has been eliminated by the binary mask, so that various defects can be effectively extracted from the mask gradient response map by iterative threshold segmentation. The operation process is as follows.

Firstly, we calculate the horizontal gradient of the original image and get the gradient response map of the Original Gradient (OG). Then, an adaptive threshold segmentation is used to get the binary image of the OG. Next, the binary image is used as a mask to eliminate the strong gradient region on the OG, thus obtaining the mask gradient response. As an iteration, we then repeat the first step on the mask gradient response map. Finally, the binary images obtained by each iteration are combined to obtain the segmentation results of different significant defects.

**Figure 5:**
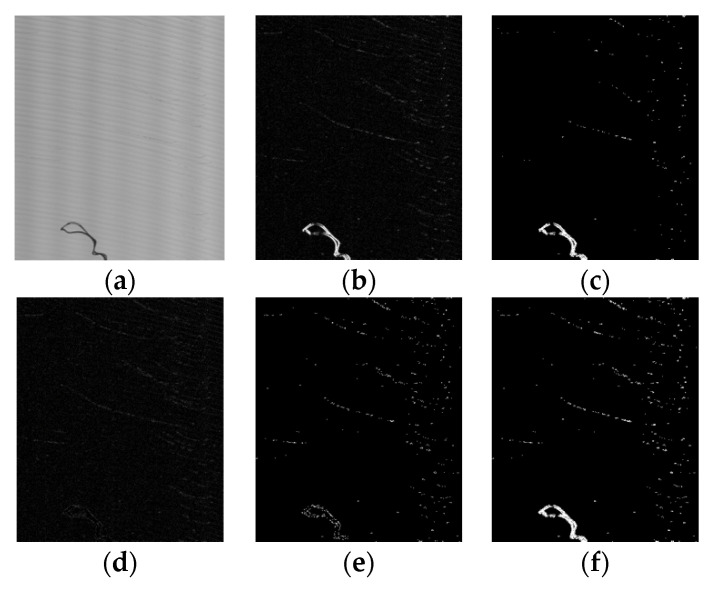
Iterative threshold segmentation of gradient map: (**a**) original image; (**b**) gradient response map (Original Gradient, or OG); (**c**) response map of the first threshold segmentation; (**d**) mask gradient (MG1) after the first threshold segmentation; (**e**) response map of the second threshold segmentation; (**f**) the final segmentation result.

As shown in Figure 5, the original image (Figure 5a) contains aluminum chips and scratches, and Figure 5b is the gradient response map based on the Sobel operator. By using iterative threshold segmentation guided by mask gradient response maps, the defect areas (Figure 5f) are segmented from the gradient map. In each iteration, adaptive threshold segmentation is realized by Equation (1) and Equation (2).

(1) $$G(x,y)=\frac{1}{2\pi \sigma {}^{2}}e^{-\frac{x^{2}+y^{2}}{2\sigma^{2}}}$$

(2) $$f_{bin}(x,y)=\{\begin{array}{c} 1,\;f(x,y)>\left[f(x,y)*G(x,y)+\lambda \sigma_{g}\right]\\ 0,\;f(x,y)\leq \left[f(x,y)*G(x,y)+\lambda \sigma_{g}\right] \end{array}$$

Equation (1) generates a Gaussian weight matrix of size m × m, where σ is the standard deviation. Equation (2) combines local Gaussian weighted sum and global standard deviation *σ_g_* to adapt to local texture changes, so that the algorithm can better extract details and improve the detection of non-obvious defects. The * denotes the convolution operator, and *λ* is the weight coefficient.

As can be seen from Figure 5, when obvious defects (aluminum chips) and slight defects (scratches) exist at the same time, the slight defects cannot be completely segmented from the gradient map after the first threshold segmentation (Figure 5c). Therefore, we adapt the iterative method and design the termination conditions. Before the second threshold segmentation, the response graph of the first segmentation is reversed to obtain the mask. The mask is applied to the gradient map to eliminate the region with strong gradient value that has been segmented in the first time, and the gradient map (Figure 5d) for the second time is obtained. Figure 5e shows the response map of the second threshold segmentation. Finally, by combining the response maps (Figure 5c, Figure 5e) of the two segmentations, the final segmentation result (Figure 5f) is obtained.

**Figure 6:**
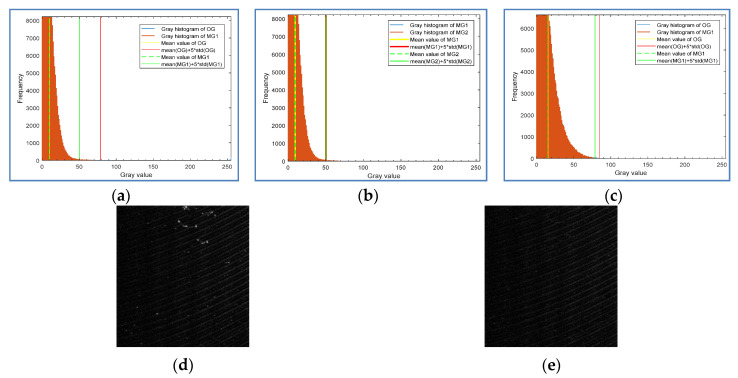
Histogram and statistical information of gradient graph in different iterations: (**a**) the original gradient (OG) and the mask gradient (MG1) after the first threshold segmentation; (**b**) the mask gradients MG1 and MG2 after the second threshold segmentation; (**c**) the OG and the MG1 of another test sample; (**d**) the OG; (**e**) the MG1.

The iteration termination conditions are made up of two parts: the maximum number of iterations and the change degree of Masked Gradient (MG). As long as one condition is satisfied, the iteration will be terminated. The maximum number of iterations is a super parameter N, and the change degree of masked gradient is calculated by Equations (3)–(5):

(3) $$g_{i}=\mathrm{mean}(MG_{i})+\lambda std(MG_{i})\;,\;i=1,2,\ldots ,N$$

(4) $$g_{i}=\mathrm{mean}(MG_{i})+\lambda std(MG_{i})\;,\;i=1,2,\ldots ,N$$

(5) $$Isover=\{\begin{array}{c} true,\;g_{i}-g_{i-1}\leq \delta \;or\;i=N\\ false,\;g_{i}-g_{i-1}\leq \delta \;or\;i<N \end{array}$$

where mean (*MG*) calculates the mean value of the masked gradient map, and std (*MG*) calculates the standard deviation. $g_{i}$ is used to describe the information distribution of the masked gradient map, and it represents the information change of the gradient map after the *i*-th iteration. When *i* is equal to 1, *MGi* − 1 = *MG*0 is the original gradient *OG*. *λ* is the weight mentioned above, and δ is the threshold of change degree. Figure 6 shows the histogram and statistical information of the gradient map in different iterations. Figure 6a shows the histogram distribution and statistical information difference of the Original Gradient (*OG*) shown in Figure 6b and the Mask Gradient (*MG*1) after the first threshold segmentation shown in Figure 5d, where *λ* is set to 5 (We first set *λ* to 3, considering that at the value of 3 sigma, the confidence probability of a normal distribution is 99.7%. To achieve better recall rate and precision, we test the value range from 3 to 3.5 with a step of 0.5. According to the test results, *λ* can be set within the range of [3.5, 5]. For relatively simple surfaces such as aluminum strip, it can be set to 3.5).

It can be observed that after the first threshold segmentation, the statistical information value *g_0_* (red solid line in Figure 6a) of *OG* is very different from that (*g*_1_) of *MG*1 (green solid line in Figure 6a), and *g*_0_ − *g*_1_ = 28.3, so the second threshold segmentation is needed. Figure 6b shows that after the second threshold segmentation, the statistical information value *g*_1_ of *MG*1 (red solid line) and the statistical information value *g*_2_ of *MG*2 (green solid line) have almost no difference, so the iteration can be ended. Figure 6c shows the distribution of statistical information of another sample after the first iteration of threshold segmentation. Figure 6d,e are the gradient map *OG* and *MG*1, respectively. For this sample, one segmentation is enough, so the distance (5.78) between *g*_0_ and *g*_1_ in Figure 6c provides a reference for the selection of the threshold.

#### 2.1.2. Difference of Gaussians

Difference of Gaussian has been well used in Scale Invariant Feature Transform (SIFT) [37] to identify potential interest points that are invariant to scale and orientation. First, the scale space of an image is defined as a function, *L(x, y, σ)*, that is produced from the convolution of a variable-scale Gaussian, *G(x, y, σ)* (defined in Equation (2)) with the input image *f(x, y)*,

(6) L(x,y,σ)=G(x,y,σ)∗f(x,y).

Then the result image of DoG can be the Difference of Gaussian function convolved with the image, *D(x, y, σ)*, which can be computed from the difference of two nearby scales separated by a constant multiplicative factor *k*,

(7) $$\begin{array}{ll} D(x,y,\sigma ) & =(G(x,y,k\sigma )-G(x,y,\sigma ))*f(x,y)\\ & =L(x,y,k\sigma )-L(x,y,\sigma ) \end{array}.$$

Considering the time consumption and defect scale, only two scales *σ* = 0 (the original image) and *σ* = 7.1 (the corresponding window size is 45) are used, and the result of DoG is

(8) D(x,y)=G(x,y,7.1)∗f(x,y)−f(x,y).

**Figure 7:**
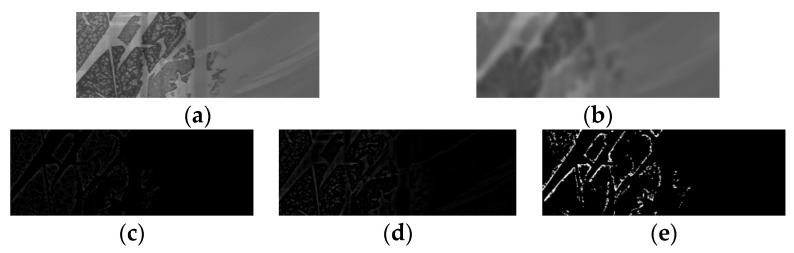
Difference of Gaussian of aluminum ingot image with a large-scale defect: (**a**) the original image; (**b**) the Gaussian blur effect; (**c**) the DoG response map calculated by Equation (8); (**d**) the DoG response map calculated by the opposite of Equation (8); (**e**) the binary image after thresholdsegmentation.

The construction of *D(x, y)* for a surface defect image of aluminum ingot is shown in Figure 7. Figure 7a is the original image with an oxide film defect. Figure 7b is the fuzzy effect image after convolution of the Gaussian function with an original image. The Gaussian window is set as 45 according to the experiment. Figure 7c is the response map of the DoG calculated by Equation (8). In the collected image of an aluminum ingot surface, the gray value of the defect area is lower than the texture background in varying degrees, so this paper uses G(x,y,7.1)∗f(x,y)−f(x,y) to reduce the influence of the background. Figure 7d shows the result of f(x,y)−G(x,y,7.1)∗f(x,y), which introduces a part of the texture response compared with Figure 7c. Figure 7e is a binary image after segmentation with a fixed threshold, and it will be combined with the result image of MGRTS by a logical OR operator.

#### 2.1.3. Similar Areas Merge

**Figure 8:**
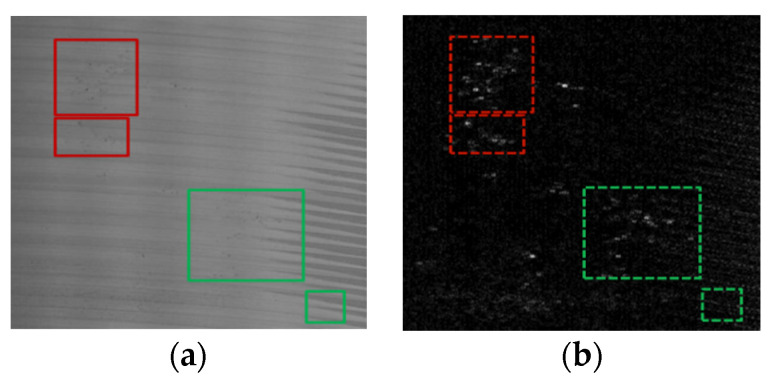
Examples of similar region (red) and dissimilar region (green) in (**a**) the original image and (**b**) the gradient image.

**Figure 9:**
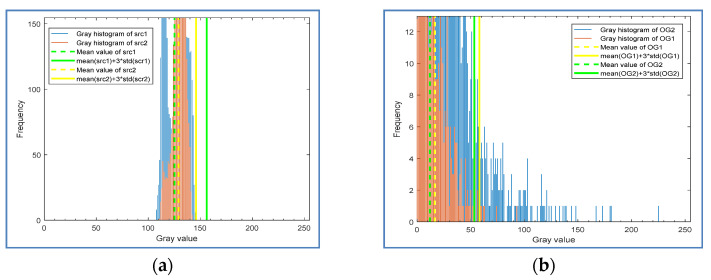
Gray histogram and statistical information of two dissimilar regions: (**a**) original images; (**b**) gradient images.

**Figure 10:**
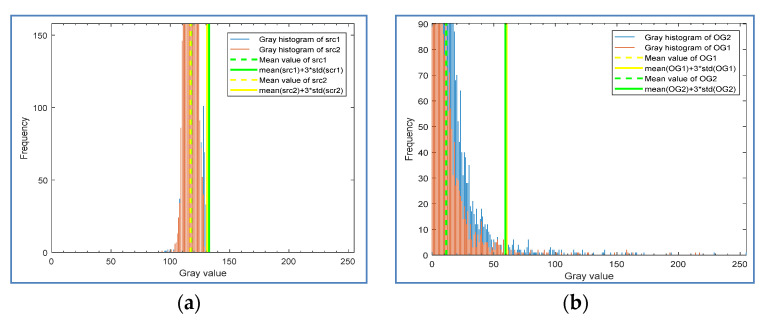
Histogram and statistical information of two similar regions: (**a**) original images; (**b**) gradient images.

The segmentation results of MGRTS and DoG are merged, and the enclosing rectangle of each defect area is obtained by contour extraction after morphological expansion. In this way, we can locate the bounding box of defects with clear boundaries, but for the distributed defects without clear boundaries, we need to further integrate the similar region, so as to obtain the bounding box of distributed defects more completely. For each defect ROI, the mean value and standard deviation of the original image (src_1_) (Figure 8a) and the gradient map (*OG*) (Figure 8b) are calculated respectively, and an information distribution descriptor v with a length of 4 is obtained. Figure 8 shows the examples of a similar region (red box) and dissimilar region (green box) in the original image and gradient image. As shown in Figure 9, for two dissimilar regions, the gray distribution histogram and statistical information (mean, standard deviation) of their original image and gradient image are different to some extent. However, for two similar regions, the difference is very small, as shown in Figure 10.

**Figure 11:**
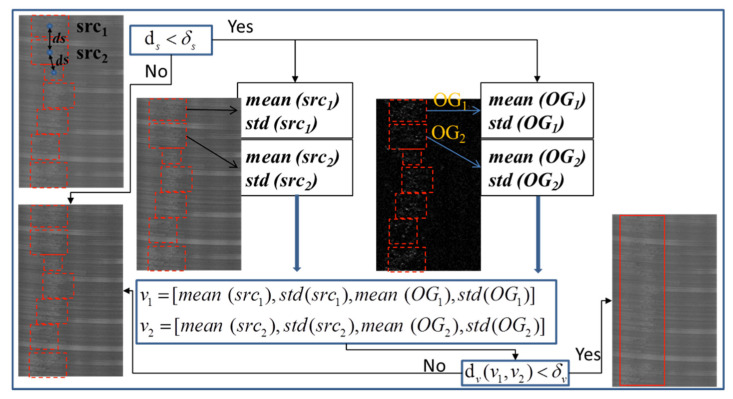
Schematic diagram of similar areas merging.

As shown in Figure 11, when iterating through the extracted candidate regions, we can decide whether to merge them into one window by calculating the spatial distance of two windows and the Euclidean distance of their information distribution descriptors.

If the two windows overlap, or the spatial Euclidean distance *d_s_* (refer to Equation (9)) between their center points *(p, q)* is very close and less than the threshold $\delta_{s}$, the information distribution vectors $v_{1}$, $v_{2}$ will be extracted, and the Euclidean distance *dv* (refer to Equation (10)) will be calculated.

(9) $$d_{s}=\sqrt{{\left(p_{x}-q_{x}\right)}^{2}+{\left(p_{y}-q_{y}\right)}^{2}}$$

(10) $$d_{v}(v_{1},v_{2})=\sqrt{\sum_{i=1}^{4}{\left(v_{1i}-v_{2i}\right)}^{2}}$$

**Figure 12:**
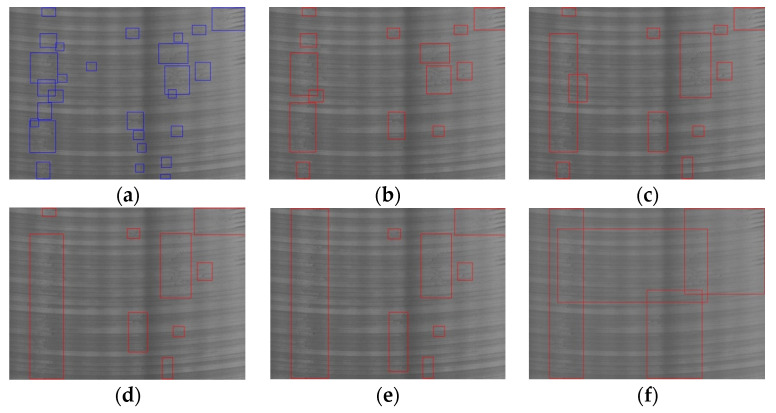
Similar areas merge results with different *d_s_*: (**a**) Before similar areas merge; (**b**) Similar areas merge result when *d_s_* is set to 30 and 40 (*d_s_* = 30, 40); (**c**) *d_s_* = 50; (**d**) *d_s_* = 60,70,80,90; (**e**) *d_s_* = 100,150; (**f**) *d_s_* = 200.

If the distance is less than the threshold $\delta_{v}$, the two windows will be merged. After testing the effect of different values on the merge results from similar areas, we set $\delta_{s}$ to 150. As shown in Figure 12, the similar areas merge results are insensitive to the value of $\delta_{s}$. For the pitted slag inclusion defect in this paper, it is better to set the threshold to 150. It is recommended to set the $\delta_{s}$ higher, as it ensures that similar areas will merge together as much as possible. Thus, defect ROIs can be completely detected, and the reduction of the ROI number will help to improve the speed of subsequent classification.

**Figure 13:**
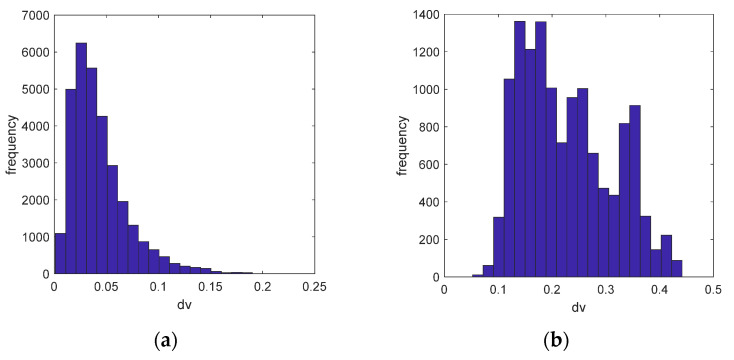
The Euclidian distance (*d_v_*) histogram of similar areas and dissimilar areas: (**a**) Similar areas distance histogram; (**b**) Dissimilar areas distance histogram.

As for the value of $\delta_{v}$, we calculate the Euclidean distance of $v_{1}$, $v_{2}$ extracted from 31,304 pairs of similar areas (Figure 13a) and 13,137 pairs of dissimilar areas (Figure 13b). As shown in Figure 13a, the profile of the histogram (H) is approximately normal distribution. According to the 3 sigma principle of normal distribution and the observation of the two histograms, we set $\delta_{v}$ = 1 (3*std(H) = 0.082).

### 2.2. Defect ROI Classification

In the classification stage, considering the strong feature extraction and representation ability of the CNN network, we use the inception-v3 [38] network structure to realize the accurate identification of various defects with large intra-class variations and high inter-class similarity.

Inception [39] is a popular convolutional neural network model proposed by Google. Its unique and detailed inception block design makes the model increase the depth and width of the network while maintaining the same amount of calculation. The inception-v3 network is the third version. The biggest change of v3 version is to decompose the 7 × 7 convolution kernel into two 1 × 7 and 7 × 1 one-dimensional convolution kernels. In this way, the calculation can be accelerated, and one convolution layer can be divided into two, which can further increase the depth of the network and strengthen the nonlinearity of the network. Since the data set of aluminum ingot surface defects used in this paper is quite different from the data set of ImageNet [40], the method of fine tune is adopted to train the model parameters.

The aluminum ingot defect samples used in this paper are collected from an aluminum ingot production line in China. The data set has the problem that the number of real defect samples is very small, and the number of false defect and texture background samples is very large. In order to overcome the class imbalance problem and improve the accuracy of defect classification, we use data augmentation technology to preprocess the sample set and introduce the focal loss into the loss function.

#### 2.2.1. Data Augmentation

**Figure 14:**
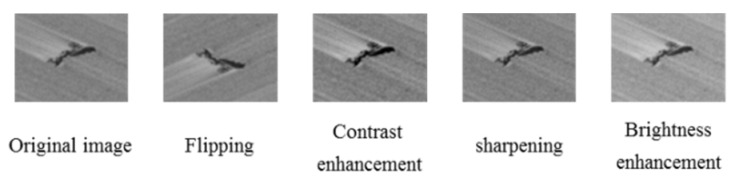
Image transformation of slag inclusion defect.

Based on the analysis of the difficult cases in online application, we use basic image transformation such as flipping, contrast enhancement, sharpening, etc. to create a larger data set. Figure 14 shows the original defect images and the corresponding transformed images.

#### 2.2.2. Focal Loss for Multi-Class

The focal loss [41] was designed by Lin et al. to address the one-stage object detection scenario in which there is an extreme imbalance between foreground and background classes during training. The focal loss for binary classification has been be given by Equation (11),

(11) $$FL(p_{t})=-{\left(1-p_{t}\right)}^{\gamma }\log(p_{t}),$$

where $p_{t}\in [0,1]$ is the estimated probability of the model for the class with label y = 1. ${\left(1-p_{t}\right)}^{\gamma }$ is a modulating factor with a tunable focusing parameter γ≥0 to down-weight easy examples and thus focus training on hard negatives. Similarly, for *k*-class classification, the formula of focal loss for multi-class (FLM) is as follows,

(12) $$FLM(P_{k\times 1})=-Y_{k\times 1}{\left(1_{k\times 1}-P_{k\times 1}\right)}^{\gamma }Y_{k\times 1}\log(P_{k\times 1}),$$

where $Y_{k\times 1}$ is a one-hot label vector with k elements and $P_{k\times 1}$ is the model’s estimated probability vector. The multiplication and logarithm here are all operations at the element level within a vector.

## 3. Results

The algorithm proposed in this paper is a two-stage target detection algorithm, so corresponding experiments are carried out to analyze and evaluate the performance of the ROI extraction and ROI classification algorithms. Finally, the performance of the whole algorithm is evaluated.

### 3.1. Evaluation Metric

In the actual production, the impact of defect missing detection is much more serious than that of false detection. Therefore, it is necessary for a surface defect detection system to have a high recall rate for real defects and ensure a high accuracy rate. In the experiments, the precision, recall, and F1-score are used to evaluate the system performance, and the accuracy is used to evaluate the classifier performance. These three metrics are defined as follows,

(13) $$precision=\frac{TP}{TP+FP},\;recall=\frac{TP}{TP+FN},\;F1\text{-}\mathrm{score}=2*\frac{precision*recall}{precision+recall},$$

(14) $$acc=\frac{TP+TN}{TP+FN+FP+TN},$$

where *TP* represents the number of true positives, *FP* represents the number of false positives, *FN* represents the number of false negatives, and *TN* represents the number of true negatives.

### 3.2. Experimental Analysis of ROI Extraction Algorithm

**Figure 15:**
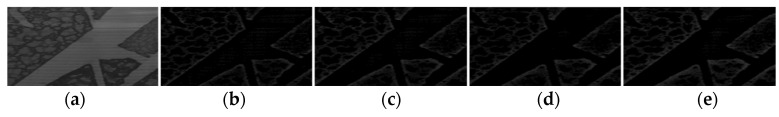
Cropped DoG results with different window sizes: (**a**) Original image; (**b**) DoG image with window size (ws) 25; (**c**) DoG image with ws 35; (**d**) DoG image with ws 45; (**e**) DoG image with ws 55.

In the MGRTS, the maximum number of iterations N and the change degree threshold are set to 5 and 6, and the Gaussian weight matrix size m in adaptive threshold segmentation is set to 25. In the DOG, we did experiments to choose the most appropriate Gaussian window size, which is related to the texture scale. We set the window sizes to 25, 35, 45, and 55 respectively to test the effect of DoG. The experiment result (Figure 15) shows that when the window size is 45, the effect is the best; that is, the DoG not only highlights the defect structure, but also suppresses most of the texture background.

**Figure 16:**
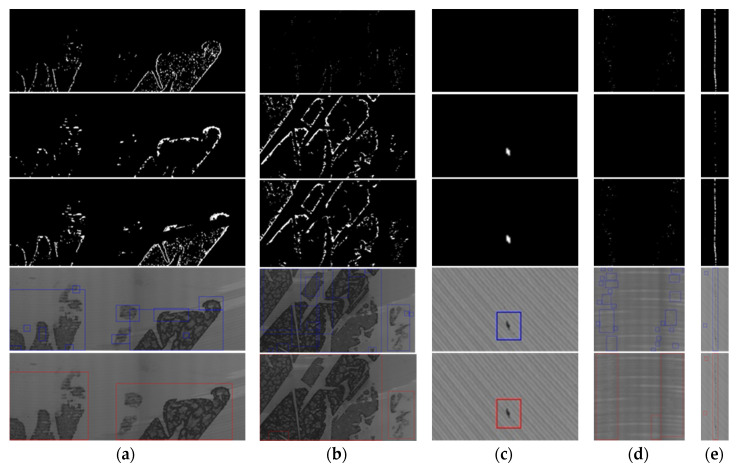
ROI extraction results of different defects: (**a**) OF; (**b**) OF; (**c**) Oil; (**d**) PSI; (**e**) Sc.

We also experimented to test the ROI extraction effectiveness of MGRTS and DoG, and the performance of the algorithm that merges similar areas. Figure 16 shows a few representative results of different defects including oxide film (Figure 16a,b), oil stain (Figure 16c), pitted slag inclusion (Figure 16d), and crack (Figure 16e). The first line of Figure 16 is the binary response map of the MGRTS, the second line is the binary response map of DoG, and the third line is the result of adding the response map of MGRTS and DoG. As shown in Figure 16b, the MGRTS failed to segment the large-scale defect completely, and MGRTS also failed to detect the oil stain in Figure 16c, which is mixed in a dense texture background, but the DoG algorithm makes up for these two disadvantages. The fourth line of Figure 16 shows the enclosing rectangle of each defect area obtained by contour extraction after morphological expansion, and the last line is the final ROI bounding box after merging similar regions. It can be seen that for large-scale defects (Figure 16a,b) and distributed defects without obvious boundaries (Figure 16d), the similar region merging algorithm can integrate local regions to obtain a complete bounding box, which also reduces the number of ROI windows and improves the classification efficiency and accuracy.

Table 1 shows the quantitative evaluation of MGRTS and DoG, and the combination of MGRTS and DoG in terms of recall and precision. We tested on a defective images data set captured in an aluminum ingot milling machine production line. The data set consists of 180 images with the size of 4096 × 1024, including 153 defects such as oxide film, oil stain, crack, slag inclusion, and pitted slag inclusion. It can be seen that the combination of MGRTS and DoG boosts the ROI extraction performance, especially the recall rate, which is more important to the production line.

### 3.3. Experimental Analysis of Defect ROI Classification

In the classification experiment, we used 32,665 defect ROI images of aluminum ingot, among which 10% sample images are selected randomly respectively as validation and training sets; the remaining 80% sample images are used as the training set. The specific number of each type of defect is shown in Table 2. It can be seen from the table that the sample number of each type of defect is extremely imbalanced.

**Figure 17:**
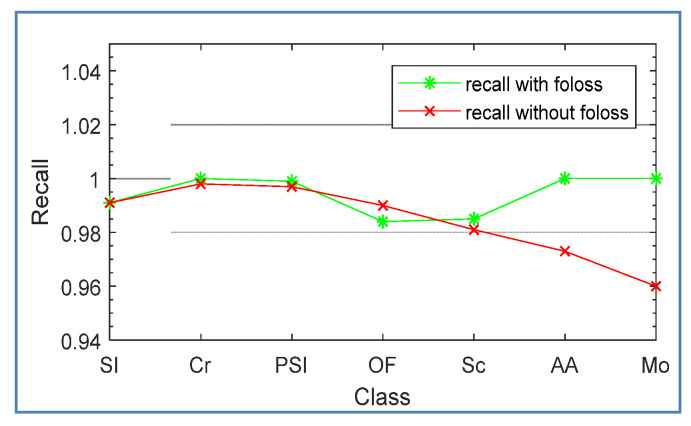
The recall curve of the two methods for each type of defect.

In order to verify the ability of focal loss to deal with sample imbalance, we compared the classification effect of using cross entropy loss and using focal loss in an inceptions-v3 network. Figure 17 shows the recall curve of the two methods for each type of defect on the test set. From the green curve in the figure, it can be seen that inception-v3 with focal loss significantly improved the recall rate of adhesion aluminum and mosquito defects with a relatively small number of samples.

We also compared the improved inceptions-v3 network with the traditional machine learning method proposed in [42]. As described in [42], we also extracted seven features including anisometry, circinal rate, ratio between the width and area, compactness, rectangularity, elongation, and ratio between area and perimeter. Furthermore, the Artificial Neural Networks (ANN) was trained with the features extracted from the aluminum ingot defect images. The classification accuracy comparison is listed in Table 3. The ANN with extracted geometric features failed to recognize the AA and Mo, because the AA defects are similar with PSI, while the Sc and the Mo defects are similar with the SI in geometry. These seven features cannot distinguish them well.

### 3.4. Overall Performance Analysis of the Proposed Algorithm

We test the overall performance of our algorithm using the database of 180 defective images described above. On the premise that the detection resolution meets the needs of the industrial field, we down-sample the image as half of the original image to improve the processing speed.

As a contrast, we also test three one-stage target detection algorithms: YOLOv3 [43], RetinaNet [41], and YOLOv4 [44]. In order to match the network structure, improve the detection accuracy, and reduce the loss of large-scale sampling, we preprocess the original annotation image. First, the original image is down-sampled to half of the original image size, and then the aluminum ingot area image after boundary detection is divided into two parts, and finally, it is normalized to 512 × 512 for network training. Due to the small sample size of the original image, we augment the defective image to three times that of the original; 2/3 of it is used as the training set, and the remaining 1/3 is used as the test set.

**Figure 18:**
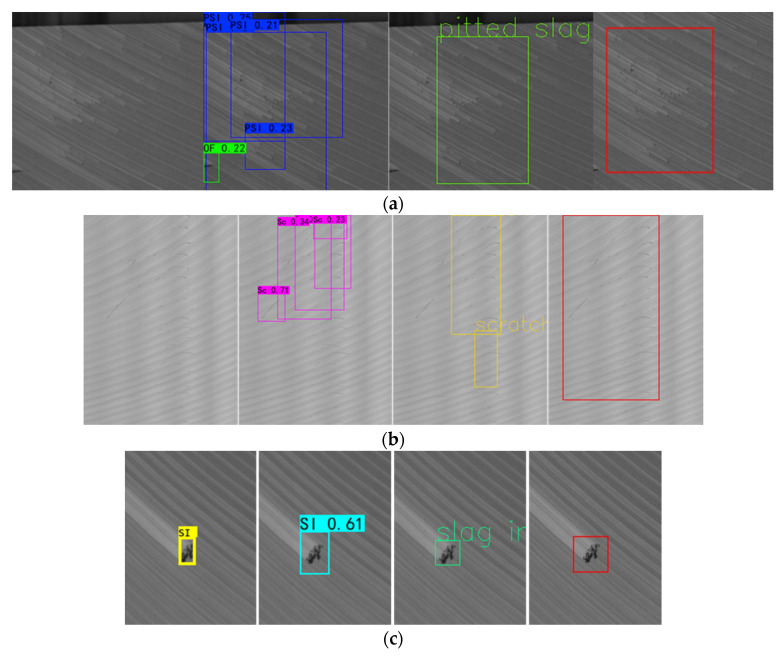

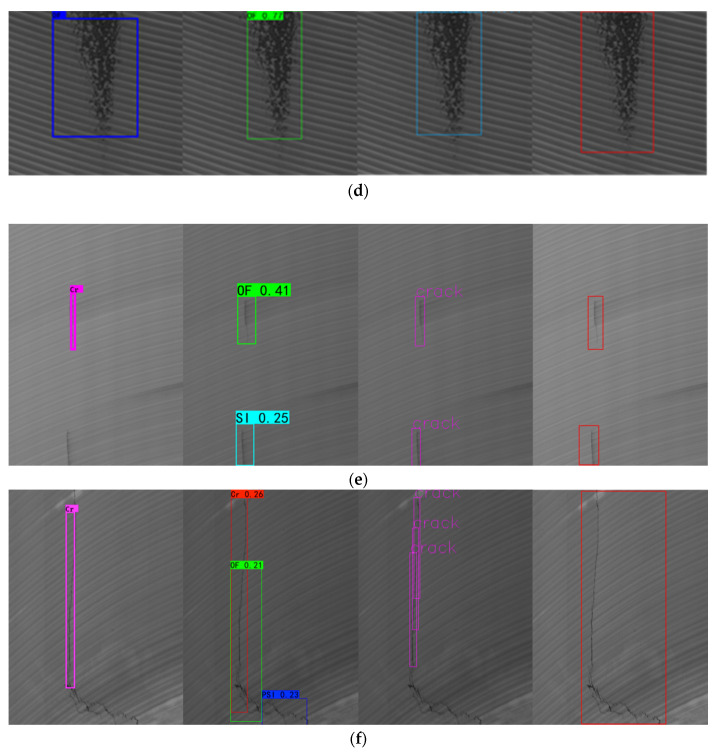

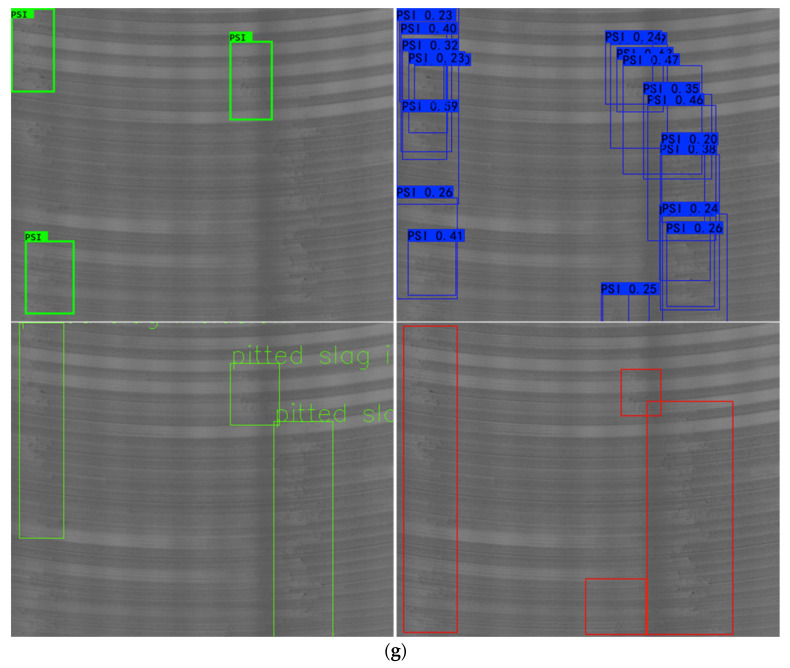
Detection effect of different methods: (**a**) PSI; (**b**) Sc; (**c**) SI; (**d**) OF; (**e**) Cr; (**f**) Cr; (**g**) PSI. The detection results of YOLOv3, RetinaNet, YOLOv4, and our method are shown from left to right in each group of comparison images.

Figure 18 shows the detection effect of the four methods for different defects. In order to prevent some small defects from being covered by the bounding boxes, we show the detection results of the four methods on four images. The detection results of YOLOv3, Retina Net, YOLOv4, and our method are shown from left to right in each group of comparison images. YOLOv3 uses multi-scale features to detect objects, and it shows a good ability to identify defects such as large-scale oxide film (Figure 18d), crack (Figure 18e,f), and small-scale slag inclusion (Figure 18c), even though the sample data set used in this paper is small. However, for the distributed pitted slag inclusion (Figure 18a,g) and the scratches (Figure 18b) with low contrast, the effect is poor, especially for the scratch defect, and the recall rate is very low. In contrast, our algorithm can detect scratches and pitted slag inclusion well because of using an iterative threshold segmentation of a masked gradient response map and the merging of similar regions.

Table 4 compares the recall (R), precision (P), and F1-score (F1) of the four methods for each type of defect. At the same time, the reasoning time of each algorithm is also listed. In experiments, the top-1 strategy was used in the statistics of detection results, and no threshold was set for the score. The average recall rate and precision of the algorithm in this paper are over 92.0%, but when influenced by a scratch defect, the average recall rate of YOLOv3 is only 66.1%. Meanwhile, RetinaNet is worse in detecting Cr defects. On the whole, the metrics of YOLOv4 are high, but similar to YOLOv3, the recall rate of scratch defects is low. Our algorithm has a relatively low recall and precision for the defects of pitted slag inclusion. The reason is that the resolution of some defects in the image is low, which affects the accuracy of defect classification. It can be seen from Table 4 that our algorithm achieves the highest F1-score and the shortest inference time.

**Figure 19:**
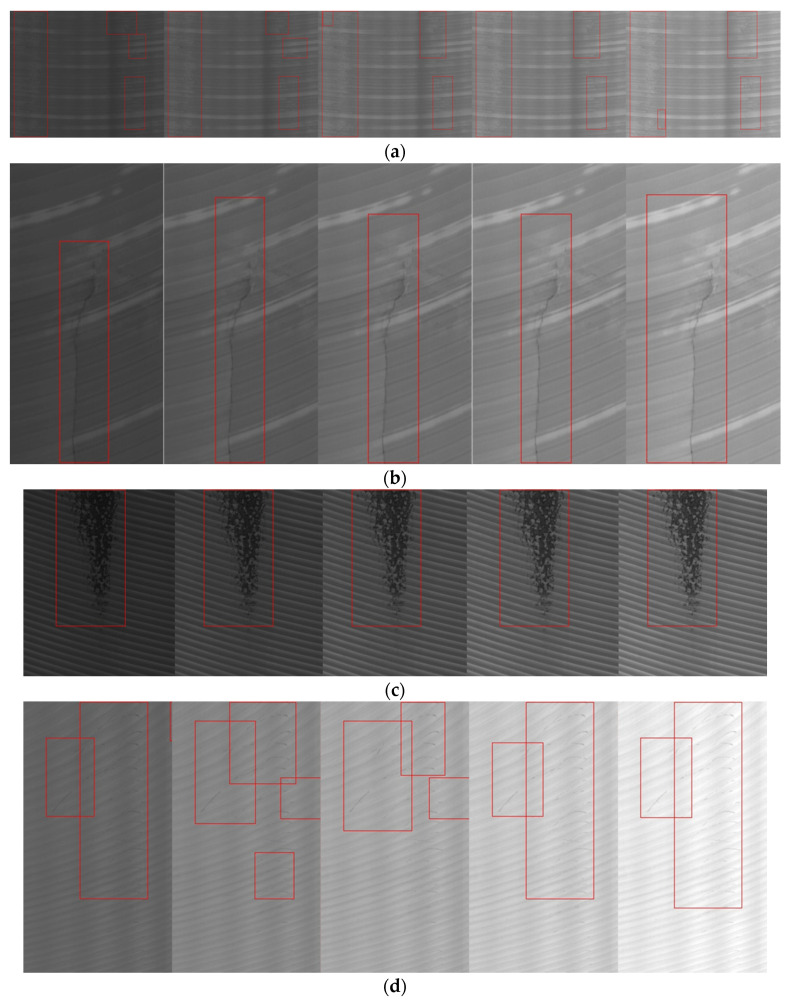
Detection effect of different brightness: (**a**) PSI; (**b**) OF; (**c**) PSI; (**d**) Sc. From left to right, the brightness of the image is reduced by 40%, reduced by 20%, unchanged, increased by 20%, and increased by 40%.

In order to test the robustness of the algorithm to illumination changes, we enhanced and reduced the brightness of the original image to simulate the change of light source brightness. As shown in Figure 19, the brightness changes of the original image are –40%, –20%, 20%, and 40%, respectively. The detection performance of the algorithm for each defect is basically not affected by illumination. This is due to the following two points: (1) ROI extraction is based on the gradient difference, which is not affected by the overall brightness change of the image. (2) As a result of the data augmentation technology mentioned in Section 2.2.1, ROI classification has a certain robustness to illumination change.

**Figure 20:**
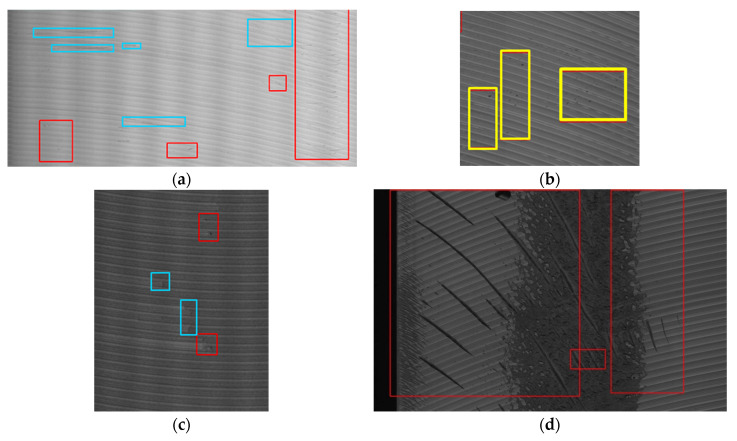
Failure cases of our method: (**a**) False negatives (blue box) of Sc (to facilitate observation, we increased the contrast by 20%); (**b**) False positives (yellow box) of PSI; (**c**) False negatives (blue box) of PSI (to facilitate observation, we increased the contrast by 20%); (**d**) Incomplete detection of OF.

We also analyzed the reason that led to the failure cases (shown in Figure 20) of our method. Figure 20a shows that our method produce false negatives of Sc defect, which are mainly caused by the low contrast and the horizontal distribution similar to the milling grain background. Similarly, some small PSI defects with low contrast are missed in Figure 20c. There were no corresponding samples in the classification network training, so the pitted oil areas are incorrectly detected as PSI defects, as shown in Figure 20b. For the large-scale oxide film shown in Figure 20d, the oxide film coverage area is too large, so that its interior is treated as regular patterns and neglected by the MGRTS +DoG. As a result, only the edge is retained.

## 4. Application in Actual Production Line

The algorithm proposed in this paper has been applied to the on-line surface defects inspection system installed at the actual production line of an aluminum ingot milling machine.

### 4.1. Image Acquisition Devices

**Figure 21:**
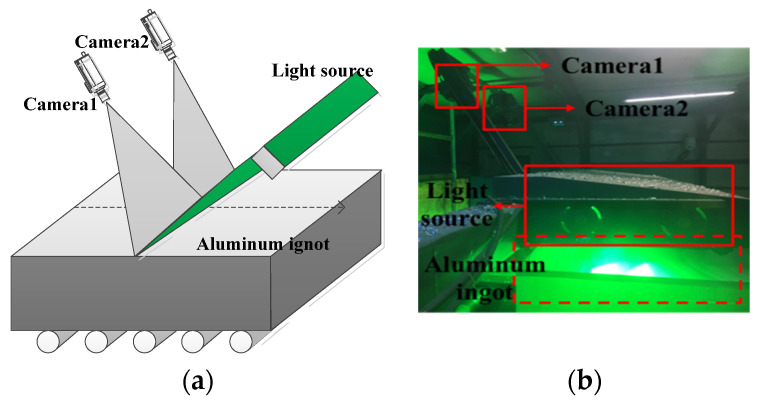
The imaging system: (**a**) a concise diagram and (**b**) picture of the material object.

Figure 21a shows a concise diagram of the imaging system, and Figure 21b is the corresponding picture of the material object. The image acquisition device includes cameras and a light source. Two line-scan charge-coupled device (CCD) cameras are used to capture images of 4096×1024 size of an aluminum ingot surface after milling under the illumination of light source, and the resolution is 0.315 mm/pixels. When the aluminum ingot passes through the acquisition device, the image acquisition program will control the acquisition speed of the camera according to the production speed and store the image. At the same time, the defect detection algorithm starts to process the image, and it alarms in time when defects are found.

### 4.2. Effectiveness of Our Method

For 5 days, we randomly checked the defect detection results of 39 production records and compared them with the real products. The detection rate of the algorithm is over 98.0%, and the accuracy of defect recognition rate is 96.0%. The statistical method of detection rate is as follows: the number of defects detected by the surface inspection system (regardless of defect category) accounts for the percentage of the number of defects on the surface of aluminum ingot.

### 4.3. Time Efficiency

The aluminum ingot region detection and the ROI extraction of the proposed approach were implemented by using C++ and OpenCV 2.4.6 library in Microsoft Visual Studio 2008, and the defect ROI classification is implemented by using python and Keras. The proposed approach was executed on a workstation with a 2.8 GHz Intel Xeon i5 processor and 16 GB memory, and the workstation is configured with a piece of NVIDIA Tesla k40c. The average time consumption of one image in each step is given in Table 5. Our detection system achieves an average processing speed of approximately 2.43 fps. The production speed of the aluminum ingot milling machine production line is from 3 to 6 m/min, when the actual production speed is 6 m/min, the corresponding camera acquisition speed is approximately 0.31 fps, so our algorithm can meet the real-time requirements.

To sum up, by applying our defect detection method to the online surface inspection system, the production is guided by the timely alarm of defects, which has great significance for ensuring product quality and improving production efficiency. In addition, the using effect also proves the promising application of our method in the surface defect detection of aluminum ingot with complex texture background after milling.

## 5. Conclusions

We proposed a novel two-stage detection approach to adaptively detect different types of defect on the surface of aluminum ingot with a complex milling grain background.

Firstly, the combination of MGRTS, DoG, and the similar region merging for the ROI extraction boosts the detection performance of various defects. Secondly, the data augmentation and the focal loss used in the inception-v3 network fine tuning handled the class imbalance well and improved the classification accuracy. Finally, the experimental results and the application in the actual production line show that when the number of defect ROI samples is large but the number of labeled original image samples is small, the performance of the two-stage defect detection algorithm proposed in this paper is significantly better than that of the one-stage deep learning algorithm. At the same time, it can also meet the real-time requirements.

Our algorithm combines the traditional detection and deep learning classification methods, which has great advantages in field application, because it can not only make full use of CPU and GPU to maximize the processing speed, but it also can be put into use quickly at the beginning of the project in the case of a lack of samples.

In future work, we will continue to collect more samples from the production line, including difficult cases and false defects. Then, we will focus on exploring a multi-scale analysis method and full convolution semantic segmentation network to further improve the detection effect of various defects.

## Figures and Tables

**Table 1:** Comparison of MGRTS, DoG, and the combination of both.

Method	Recall	Precision
MGRTS	97.4%	56.2%
DoG	54.9%	93.3%
MGRTS + DoG	99.3%	56.7%

**Table 2:** Specific of each type of defect used in the classification experiment.

Defects	SI	PSI	Cr	AA	Sc	OF	Mo	Tb	Total
Total	3709	5413	5204	347	1932	6062	902	9096	32,665
Train	2969	4331	4164	249	1546	4850	722	7278	26,139
Validation	370	541	520	34	193	606	90	909	3263
Test	370	541	520	34	193	606	90	909	3263

**Table 3:** Classification accuracy of Artificial Neural Networks (ANN), inceptions-v3, and inceptions-v3 with focal loss.

Defects	SI	PSI	Cr	AA	Sc	OF	Mo	Average
ANN	83.0%	89.0%	84.0%	0.0%	59.0%	42.0%	0.0%	51.0%
inceptions-v3	99.1%	99.9%	100.0%	97.3%	98.1%	99.0%	99.6%	99.0%
inceptions-v3 with focal loss	99.1%	99.7%	99.8%	100.0%	98.5%	98.4%	100.0%	99.4%

**Table 4:** Performance comparison of the four methods for each type of defect.

Method	Our Method			YOLOv3			Retina Net			YOLOv4		
Metric	R	P	F1	R	P	F1	R	P	F1	R	P	F1
	(%)			(%)			(%)			(%)		
Sc	96.7	93.8	95.2	3.6	66.7	6.8	71.4	93.0	80.8	75.9	100.0	86.3
OF	95.7	98.2	96.9	90.5	93.5	92.0	87.4	86.5	87.0	88.4	97.9	92.9
Cr	98.6	98.6	98.6	88.9	92.3	90.6	15.9	81.3	26.5	100.0	98.8	99.4
SI	94.1	91.9	94.0	76.9	69.0	72.7	57.7	62.5	60.0	84.6	100.0	91.7
PSI	88.6	85.2	86.9	70.3	95.5	81.0	91.9	85.3	88.5	82.8	98.7	90.1
Average	94.7	93.5	94.3	66.1	83.4	68.6	64.8	81.7	68.5	86.3	99.1	92.1
Time (ms) 512 × 512	103			233			304			167		

**Table 5:** Average time consumption of one image in each step.

Process Stage	Time Consumption (ms)
Aluminum ingot region detection + ROI extraction	272
Defect ROI Classification	140
Total	412
